# *Escherichia coli* antimicrobial resistance in acute urinary tract infection lower than reported in Dutch national surveillance database

**DOI:** 10.1371/journal.pone.0334222

**Published:** 2025-10-09

**Authors:** Marjan J. Bruins, Linda Eijkelkamp-Biesterbos, Annemiek M. Meutstege, Claudy Oliveira dos Santos

**Affiliations:** 1 Laboratory of Clinical Microbiology and Infectious Diseases, Isala hospital, Zwolle, The Netherlands; 2 General Practice Zuyderhart, Zwolle, The Netherlands; 3 General Practice Dok Zuid, Zwolle, The Netherlands; Hawassa University College of Medicine and Health Sciences, ETHIOPIA

## Abstract

**Background:**

General practice patients with an acute urinary tract infection are usually treated empirically. Urine is only cultured if initial therapy fails. This study aimed to assess whether resistance rates of *Escherichia coli* (*E. coli*) from initial urine samples differ from those observed after treatment failure, and whether current surveillance data used for treatment guidelines may overestimate resistance in first-line care.

**Methods:**

A prospective observational study was conducted from October 2022 to June 2024 at Isala Hospital in Zwolle, the Netherlands, involving patients from two general practices. Patients with suspected acute UTI were included, excluding those with indwelling catheters. Initial urine samples were cultured, and resistance rates for all *E. coli* strains isolated were compared with data from the Dutch national AMR database ISIS-AR and regional data. Treatment success or failure was evaluated by recording the prescribed antibiotics and follow-up visits within 28 days.

**Results:**

A total of 366 urine samples from 272 patients were analyzed. *E. coli* was the most common pathogen (82.2%). Resistance rates for *E. coli* were lower than those reported in national and regional datasets. Empirical treatment success rates were 95% for nitrofurantoin, 90% for fosfomycin, 92% for ciprofloxacin, and 100% for trimethoprim. Repeat cultures were performed in 19 cases due to persistent symptoms.

**Conclusion:**

The study confirms that the Dutch national guideline for empirical UTI treatment in general practice is effective. However, periodic reviews of resistance rates for common uropathogens are recommended to ensure continued guideline validity and promote responsible antibiotic use.

## Introduction

Urinary tract infections (UTIs) are common in the general practice. In 2023, the incidence of cystitis/UTIs in Dutch general practices was 117.2 per 1000 women and 20.4 per 1000 men, accounting for 2.7% of all consultations [1].

When acute UTI is suspected in a general practice patient, usually empirical treatment is started according to the national guideline for UTI [2]. While this treatment is usually effective, if symptoms persist, a second and sometimes even third course of antibiotics may be prescribed without taking a urine culture.

UTI guidelines recommend sending a urine sample to the microbiological laboratory for culture and antimicrobial susceptibility testing only after one or more instances of therapy failure. This suggests that the ultimately cultured and reported infectious agents could theoretically be more resistant than the bacteria that caused the initial, untreated UTI.

Resistance patterns of cultured microorganisms are stored in the Dutch national antimicrobial resistance (AMR) database, which is used, among other things, to develop guidelines for the empirical antibiotic treatment of infectious diseases. The potential bias resulting in reporting too high resistance rates has been noted elsewhere, but has not yet been investigated in the Netherlands [3–6]. To determine whether the resistance rates on which the UTI treatment guideline is based might be overestimated for UTI pathogens, a study was set up in collaboration with two general practices in our laboratory service area. Contrary to what the UTI guideline indicates, of all patients suspected of an acute UTI a urine sample was sent for culture immediately, before prescribing the first empirical treatment.

In this prospective study, we aimed to identify possible differences between these local findings and regional and national resistance rates, specifically for *Escherichia coli* (*E. coli*) as the most frequent UTI pathogen. We also assessed whether the empiric therapy recommended in the UTI guideline should be adjusted in any way based on the observed resistance.

## Materials and methods

### Setting, sample collection and processing

The Laboratory of Clinical Microbiology and Infectious Diseases of Isala Hospital in Zwolle provides infectious disease diagnostics for over 250 general practitioners (GPs) in the northeast of the Netherlands. The study was conducted from October 2022 to June 2024, involving patients from two general practices in Zwolle. All patients suspected of acute UTI according to the Dutch College of general Practitioners (NHG) UTI guideline were included [2]. An acute urinary tract infection is an infection of the urinary tract characterized by the sudden onset of symptoms such as painful or burning urination, frequent urge to urinate, urgency without actual urine production, and possibly blood in the urine [2]. Patients with an indwelling catheter were excluded as well as urine samples sent for treatment control purposes. Patients were included more than once if they presented with a new UTI episode at least 28 days after their previous visit during the study period.

The general practitioners in the two participating practices follow this guideline: when a UTI is suspected, a medical history is taken and, if necessary, a physical examination is performed. Preferably, the first morning void of (midstream) urine is tested for nitrate and leukocytes. If the urine test strip results are inconclusive, a dip slide test is performed to confirm bacterial growth. Treatment is then prescribed blindly according to the guideline. In most cases, no initial urine sample is sent to the laboratory for culture.The guideline recommends culturing only after one or more therapy failures, unless the patient belongs to a special category: elderly, child, pregnant, immunocompromised, etc., or if tissue invasion is suspected. For our study, between October 11^th^, 2022, up to and including June 11^th^, 2024, of each patient, the first urine sample was sent to our laboratory and cultured according to standard laboratory procedures. All samples were processed as follows: urine was cultured on a set of selective agar plates and a Gram stain is made. Bacterial growth is evaluated based on potential uropathogens in relation to the quantity of urogenital flora and the number of leucocytes. Isolates were identified with Matrix-assisted laser desorption ionization-time of flight mass spectrometry (MALDI-TOF; Bruker Daltonik, Bremen, Germany), antimicrobial susceptibility testing (AST) was performed using the Vitek 2 system (bioMérieux, Marcy-l’Ėtoile, France) with EUCAST breakpoints. Culture results were reported to the GP as usual.

The local Medical Research Ethics Committee considered the study not subject to the Dutch Medical Research Involving Human Subjects Act (WMO) and declared it to be exempted from further review. No consent from the participants was needed.

### Data comparison and follow up

Resistance percentages were calculated for all *E. coli* strains isolated from the included cultures for the oral antibiotics ampicillin/amoxicillin, amoxicillin-clavulanic acid, cefuroxime axetil, ciprofloxacin, fosfomycin, nitrofurantoin, trimethoprim, trimethoprim-sulfamethoxazole and third generation cephalosporin cefotaxime/ceftriaxone. Of these, the oral agents nitrofurantoin, fosfomycin and trimethoprim are the most commonly used to treat acute UTI. Pivmecilliman is currently being investigated in the Netherlands and is not (yet) used as a first-line treatment for UTI.

The observed local resistance rates were compared with data from 2023 in the Dutch national AMR database Infectious Disease Surveillance Information System for Antibiotic Resistance (ISIS-AR, accessed *at* www.ISIS-web.nl in January 2025), where national AST data sent in by most Dutch clinical microbiology laboratories are registered by the National Institute for Public Health and the Environment (RIVM) [7]. Querying the database provides data on the first entered isolate per species per patient per calendar year. If two *Escherichia coli* isolates with different resistance patterns were cultured from one sample, the first isolate is selected.

Comparison was also made with regional data from the same period as from the two participating general practices, i.e., obtained from all other general practices in the region that consistently submit urine samples to our laboratory and of which identification and AST data are stored in our laboratory information system (LIS). The same selection method as was used for the national database was applied here. After data collection and analysis, the data could not be traced back to individual participants.

Demographic data comprising participants’ age and sex were recorded. Differences in resistance rates were calculated using SPSS version 28, with a p-value < 0.05 considered statistically significant.

To assess treatment success or failure we recorded which antibiotic was empirically prescribed after the first urine sample was collected, as well as the frequency and, if possible, the reason of patient return visits within 28 days thereafter. Treatment failure was defined as microbiological, i.e., the cultured uropathogen was found to be resistant against the administered antibiotic, or as clinical: the findings indicated colonization and no true UTI, or the case involved a complicated UTI for which the treatment proved ineffective.

## Results

### Culture results

In total 366 first urine samples were collected from 272 patients (243 female (89%), 29 male), with a median age of 57 years (range 1–98 years). Cultured uropathogens are shown in Table 1. Mixed flora (≥ 3 species) was reported in 19 cultures (5.2%), in 27 cultures (7.4%) urethral flora only, and in one sample (0.3%) no growth was found. *E. coli* was the most frequently isolated pathogen (82.2%), followed by *Staphylococcus saprophyticus* and *Klebsiella pneumoniae*.

**Table 1:** Uropathogens cultured from first urine samples.

Species	N	%
*Escherichia coli*	273	82.2
*Staphylococcus saprophyticus*	14	4.2
*Klebsiella pneumoniae*	13	3.9
*Enterobacter cloacae*	5	1.5
*Klebsiella oxytoca/Raoultella* species	5	1.5
*Proteus mirabilis*	4	1.2
*Aerococcus urinae*	3	0.9
*Citrobacter koseri* group	3	0.9
Group B beta-hemolytic streptococci	3	0.9
*Pseudomonas aeruginosa*	2	0.6
*Serratia marcescens*	2	0.6
*Staphylococcus aureus*	2	0.6
*Actinotignum* species	1	0.3
*Citrobacter freundii* group	1	0.3
*Serratia liquefaciens*	1	0.3
Total	332	

### Resistance rates of *E. coli*

Resistance rates of the 273 *E. coli* study isolates were compared to regional percentages (10,872 isolates) and to national percentages (approximately 138,000 isolates), Table 2. National ISIS-AR database resistance rates for amoxicillin/ampicillin, cefuroxime, ciprofloxacin, fosfomycin, trimethoprim and trimethoprim-sulfamethoxazole were significantly higher than those found in the study of isolates recovered from first urine samples. Regional rates were higher for amoxicillin/ampicillin and ciprofloxacin compared to the study rates.

**Table 2:** *E. coli* resistance rates.

	Resistance rates (%)			Fisher’s Exact Test (P value)	
Antibiotic	Study	National	Regional	Study vs. National	Study vs. Regional
Amoxicillin/ampicillin	28.2	34.5	34.2	0.031	0.044
Amoxicillin-clavulanic acid	23.4	24.6	26.5	0.271	0.266
Cefotaxime/ceftriaxone	2.6	4.3	3.5	0.179	0.643
Cefuroxime axetil	4.8	8.2	7.6	0.041	0.081
Ciprofloxacin	3.7	9.0	8.6	0.002	0.015
Fosfomycin	0.4	2.1	1.6	0.035	0.135
Nitrofurantoin	0.4	1.4	1.8	0.195	0.096
Trimethoprim	15.0	20.5	19.0	0.026	0.100
Trimethoprim-sulfamethoxazole	12.5	18.1	17.0	0.017	0.141

### Therapy outcome and follow up

Of the 272 participating patients, for 256 patients no follow-up cultures were submitted within 28 days after the first urine sample. Of these, 207 patients were registered with one UTI episode, others with two (n = 36), three (n = 13), four (n = 3) and five (n = 1) recurring UTIs. This total of 335 episodes were apparently successfully empirically treated with nitrofurantoin (n = 245), fosfomycin (n = 30), ciprofloxacin (n = 24), trimethoprim (n = 8), amoxicillin (n = 5) and amoxicillin/clavulanic acid (n = 4). Fourteen suspected UTIs were not treated and in five cases the treatment was unknown.

In 19 cases (18 patients), a repeat urine sample was cultured within 28 days after the first sample because of persistent symptoms consistent with a UTI. Of the 13 patients empirically treated with nitrofurantoin, one patient had a culture with a nitrofurantoin resistant *Klebsiella pneumoniae*. For the other twelve patients the reason of treatment failure was unknown. Unexplained treatment failure also occurred after prescription of fosfomycin (n = 3) and ciprofloxacin (n = 1). One patient who was initially treated with ciprofloxacin was admitted immediately with an urosepsis, which was subsequently treated successfully with ceftriaxone. One patient who did not receive treatment at the first visit kept complaints, which were successfully treated with nitrofurantoin prescribed at the second visit.

Successful empirical treatment with nitrofurantoin was achieved in 245 of 258 cases (95%). Success rates for fosfomycin, ciprofloxacin and trimethoprim were 90% (27/30), 92% (24/26), and 100% (8/8) respectively.

## Discussion

The three main uropathogens cultured from the 366 study first urine samples from patients suspected of an acute UTI were *E. coli*, *Klebsiella pneumoniae* and *Staphylococcus saprophyticus*, with prevalence consistent with existing literature [8–10].

Resistance rates for a number of antibiotics for 273 isolates of *E. coli* cultured from first urine samples were as expected lower than those registered in the Dutch national ISIS-AR database. The largest differences were found for ciprofloxacin, also when compared to regional rates, and for trimethoprim-sulfamethoxazole. This can be explained by the selection bias in the ISIS-AR data due to culturing only after blind empiric therapy failure. Most of the first urine samples in our study were associated with acute, uncomplicated UTIs, whereas the national database includes data from all cultures, including those from complicated infections. This suggests that the expected bias toward higher resistance rates was confirmed. The same holds for comparison with regional rates, as usually no first urine samples are sent in.

A limitation of the study was the relatively low number of *E. coli* isolates compared to the large volume of data in the ISIS-AR database. This indicates that further research is desirable to more clearly show observed differences.

In total 335 acute UTIs were most frequently treated with nitrofurantoin, fosfomycin and trimethoprim, showing good adherence to the UTI guideline. Ciprofloxacin was prescribed in 24 cases, mostly because of symptoms of fever or known allergies. Therapy success for these antibiotics ranged between 90–100%.

We included all patients with an acute urinary tract infection (UTI) in our study, except those with an indwelling catheter. Some patients already had an invasive infection at their initial presentation, as evidenced by the clinical data. They were also treated according to the national guideline.

In the national database ISIS-AR, only the first isolate per species from a culture is stored, based on the sequence number assigned to the isolates in the laboratory. The choice of a more or less resistant strain is therefore random. However, due to the large numbers, this will not affect the outcomes of the comparisons. The same applies to the large number of cultures from the region for which differences in resistance percentages have been calculated.

Therapy failure can occur when the microorganism being targeted is resistant to the given treatment (microbiological failure). In our study, we observed this only once, with a nitrofurantoin-resistant *Klebsiella pneumoniae*. In follow-up cultures from patients who returned with persistent symptoms, we also observed a more resistant strain of *E. coli* once. However, since in this study the number of returning patients was small, there is little to conclude from this. More and longer research could provide greater insight into microbiological failure. In one case of UTI caused by *Klebsiella pneumoniae* blind therapy with fosfomycin failed, probably because the effectiveness of fosfomycin against *Klebsiella* spp. can be limited [11].

Clinical therapy failure despite a susceptible pathogen can occur because the required antimicrobial concentration is not high enough in the bladder or surrounding tissues of the urinary tract (e.g., bladder wall, kidney) in the case of a more complicated UTI and f.i. nitrofurantoin does not reach tissue levels. There may be no actual urinary tract infection despite complaints (only urethral flora cultured), or there is contamination with fecal matter (mixed flora in the culture). Other abnormalities in the urinary tract may also be causing the symptoms. Non-compliance with therapy is also possible if patients stop the treatment as soon as symptoms rapidly subside. Further research into the exact backgrounds of therapy failure would be beneficial.

Others have performed smilar studies on specifically *E. coli* in urine cultures of outpatients, with differing rates of resistance as a result [10,12]. Recommendations in guidelines for the initial empirical treatment of infectious diseases, specifically for urinary tract infections (UTIs), should primarily be based on epidemiological data [2,10,13,14]. Worldwide, the resistance percentage thresholds vary, and there is no uniform method for determining them [10,15]. If resistance increases, thresholds should be adapted. Our results indicate that the first-choice agents according to the Dutch national guideline are adequate, and adjustments are not necessary at this time. Previous studies addressed the question whether national resistance rates may underestimate antimicrobial susceptibility, resulting in too broad-spectrum therapy and possible resistance development [3–5,16]. The actual resistance percentages found for the initial urine samples in our study correspond with those in the German study of Klingeberg et al. and overall conclusions are similar to ours [17].

In most cases of UTIs, the empirical therapy according to the national UTI guideline is adequate. However, periodically review of the resistance percentages for the most common uropathogens in acute UTIs is advisable, in order to continue validating the guidelines.
